# Immunogenicity of native and CD4 liganded monomeric and trimeric envelope glycoproteins based on HIV-1 Subtype C consensus Founder virus sequences

**DOI:** 10.1186/1742-4690-9-S2-P21

**Published:** 2012-09-13

**Authors:** MA Killick, A Capovilla, MA Papathanasopoulos

**Affiliations:** 1 HIV Pathogenesis Research Laboratory, Johannesburg, South Africa

## Background

The ability to induce a broadly neutralizing antibody (bNAb) response following vaccination is regarded as a crucial aspect in developing an effective HIV-1 vaccine. This study describes the design and construction of a subtype C founder virus consensus Env immunogen derived from newly transmitted/founder virus sequences, and its immunogenicity testing in the presence or absence of liganded CD4, in small animals.

## Methods

Monomeric (gp120), dimeric (gp120GCN4) and trimeric (gp140GCN4 +/-) founder virus conformations were expressed in mammalian cell culture. Unliganded or 2dCD4^S60C^ liganded Env glycoproteins were purified by lectin affinity chromatography, followed by conformation and complex purification using size exclusion chromatography. Immunogens/immune complexes were evaluated by ELISA, SDS-PAGE, Native PAGE and Surface Plasmon Resonance. Immunogenicity of each conformation alone or complexed to 2dCD4^S60C^ was evaluated in rabbits. Breadth and potency of the rabbit sera was tested against 12 pseudoviruses (Tiers 1-3), derived from HIV-1 subtype B and C Env, using the PhenoSense Neutralizing antibody assay (Monogram Bioscience Inc.).

## Results

Minimal neutralizing breadth was obtained from animals immunized exclusively with Env conformations. However, animals that received the Env/2dCD4^S60C^ complex showed extensive neutralizing capacity against all 12 viruses tested, including the tier 2 and 3 virus strains. End-point ELISA titre results revealed that the rabbits that were immunized with Env/2dCD4^S60C^ produced both Env and 2dCD4 specific titres, but those directed towards 2dCD4 were on average 10x lower than the 2dCD4 control group. This implies a proportion of the neutralizing antibody activity is directed towards conserved epitopes exposed on the Env/2dCD4^S60C^ immunogens.

## Conclusion

The ability to induce bNAb activity in previous immunization studies utilizing Env/CD4 complexes was attributed to the induction of high anti-CD4 titres. By contrast, in our study the relatively low anti-CD4 titres compared to anti-Env titres and neutralization profiles suggest an alternative mechanism of neutralization other than a response directed to CD4 alone.

